# Adult stakeholders' perspectives on supporting or undermining the mental health of sexual and gender minoritised adolescents

**DOI:** 10.1111/papt.12548

**Published:** 2024-10-05

**Authors:** Rajvinder Samra, Mathijs F. G. Lucassen, Alicia Núñez‐García, Katherine E. Brown, Katharine A. Rimes, Louise M. Wallace

**Affiliations:** ^1^ Department of Health and Social Care School of Health, Wellbeing and Social Care, The Open University Milton Keynes UK; ^2^ Department of Nursing School of Health and Psychological Sciences, City St George's, University of London London UK; ^3^ Public Health and Applied Behaviour Change Laboratory (PHAB Lab) School of Life and Medical Sciences, University of Hertfordshire Hatfield UK; ^4^ Department of Psychology Institute of Psychiatry, Psychology and Neuroscience, King's College London London UK

**Keywords:** adolescents, adult perspectives, gender, gender minority, LGBTQ+, mental well‐being, sexual minority, sexuality, support

## Abstract

**Objectives:**

To explore adult stakeholders' perspectives on what supports or undermines the mental health of sexual and gender minoritised adolescents (SGMA) in everyday life in order to better understand how to foster supportive psychosocial environments for SGMA.

**Design:**

Descriptive qualitative study design, using framework analysis.

**Methods:**

Semi‐structured interviews were conducted remotely with 16 UK‐based adult stakeholders which included parents of SGMA, health and social care professionals, community‐based professionals, and professionals who commission services related to adolescent health and well‐being.

**Results:**

Nine themes were identified that represented barriers and enablers of fostering psychosocial environments that are supportive of SGMA mental health. Example barrier themes include SGMA ‘facing chronic and acute safety threats and stress’, ‘psychological responses to social connection losses and navigating alienation’, ‘digital exposure and online risk and vulnerability’ and ‘conflicting messages, resulting divisions and adult distancing’. Example enablers include ‘exploring, owning, and changing (personal) identities’, ‘advocating alongside adolescents whilst containing oneself as the adult in the situation’ and ‘personally fostering adolescents’ psychological safety and inclusion’.

**Conclusions:**

Adult stakeholders report that SGMA are often exposed to environments hostile to key aspects of their identity which then by extension undermines their mental health. These experiences can threaten their sense of safety and evolving identity. Practitioners in particular should be aware of the stressors relating to SGMA identity and minoritisation experiences in order to develop the psychological safety and sense of inclusion needed for SGMA to trust in the relationship and the support offered.

## INTRODUCTION

Sexual and gender minoritised adolescents (SGMA) include young people who identify as lesbian, gay, bisexual, transgender/trans and queer, as well as all other sexual and gender minoritised (i.e. LGBTQ+) individuals. The World Health Organization defines adolescence to be between the ages of 10 and 19 years old (WHO, 2021). However, we acknowledge this rather restrictive definition is contested, for instance, by Sawyer et al. (2018) who argue that the period between 10 and 24 years aligns better with contemporary patterns of adolescent development, as well as popular conceptions of this life stage. It is well established that SGMA experience elevated rates of mental health problems such as depression, suicidality and self‐harm (e.g. Lucassen et al., 2017; Marchi et al., 2022; Williams et al., 2021). SGMA are also at greater risk of social rejection, including from their school peers, teachers and their own family members; this carries additional consequences (alongside mental distress), such as avoiding or changing schools, homelessness (Ormiston, 2022; Strauss et al., 2017), becoming disengaged from their education and consequently struggling with educational achievement (Day et al., 2018; Fenaughty et al., 2019).

Subgroups within the wider SGMA population also indicate differential rates of mental distress, stigma processes and other related experiences. For instance, there is some indication of elevated rates of suicidality and self‐harm amongst bisexual (Dunlop et al., 2020; Lucassen et al., 2011) and transgender/trans people (e.g. Liu et al., 2019; Pompili et al., 2014; Wittgens et al., 2022) in comparison to their lesbian and gay cisgender (i.e. someone whose gender identity corresponds to the sex they were assigned at birth) counterparts. Rimes et al. (2019) also reported important differences in the experiences of transgender adolescents who identify within, versus outside of, the gender binary (e.g. non‐binary) of girl or woman/boy or man. Whilst mental health problems are more prevalent in SGMA than in their heterosexual cisgender adolescent peers, varied experiences and manifestations of this distress, within and across the wider SGMA umbrella, need to be explored.

Importantly, a significant proportion of the adverse and hostile experiences encountered by SGMA will be driven – deliberately or unintentionally – by the key adults in the young person's life. For example, discrimination from health professionals represents an important barrier to accessing health care when this is needed and contributes to further social rejection amongst SGMA (Town et al., 2022). Parental/caregiver and family rejection can also lead to exclusion from one's home and/or childhood social and cultural communities (Salerno et al., 2022). At school, trans young people report experiencing insensitive language and treatment related to their gender identity from teachers and school‐based professionals as well as their peers (Zeeman et al., 2017). Furthermore, school‐based professionals may struggle with responding appropriately when young people experience transphobic, biphobic and homophobic victimisation (Zeeman et al., 2017).

Minority stress theory posits that societal reactions to one's identity can result in distal stress from external actions such as bullying, and proximal stress from within the person, such as the internalisation of transphobic, biphobic and homophobic messages (e.g. Meyer, 2003; Moyano & Sánchez‐Fuentes, 2020; Williams et al., 2023). SGMA, like all young people, can lead complex private and public lives within online and offline contexts. Relatedly, SGMA must also manage the risks linked to living amongst society's pervasive cisgender and heteronormative pressures, across environments and contexts where there are threats of family or school‐based rejection (Cooper & Blumenfeld, 2012).

Some aspects of common structural stressors for SGMA may make them isolated and in turn, place them at an increased risk of mental distress. For example, in a study of SGMA (aged approximately 10 and 16 years), participants reported greater use of online communities than their peers, to help them feel less alone and because they had no one to talk to about their sexual orientation (and presumably for some their gender identity) (Charmaraman et al., 2021). Although online contact can garner support, it may not be as effective as face‐to‐face social contact and it can also expose users to unsafe environments, including more prejudice and victimisation (Lucassen et al., 2018). Some social and cultural exclusions, plus barriers, place SGMA at greater risk of mental distress and it is pertinent to explore adults' viewpoints about how to tackle such barriers. In family and school environments, adults can play important roles in supporting or trying to control young people's use of online as well as in‐person environments. In society more generally, adults can express attitudes (or create content) that young people might consider discriminatory or oppressive (as well as the opposite), but these serve to illustrate the relevance of seeking the viewpoints of adults. The research evidence base would therefore benefit from the views of key adult stakeholders, such as school‐based practitioners, parents/caregivers (henceforth, ‘parents’) and mental health professionals to help bring about wider system changes for the benefit of SGMA (Zeeman et al., 2017).

A key advantage of engaging with adult stakeholders, relevant to the lives of SGMA, is the information that they can provide about the categories of SGMA who are likely to be systematically and structurally disadvantaged in engaging in adolescent‐focused research. For example, in a study investigating the under‐involvement of SGMA in adolescent‐focused sexual health research, Macapagal et al. (2017) noted that participants can fear being ‘outed’, particularly when their family is perceived as less accepting, for reasons such as religious beliefs and affiliations. Additionally, the authors found that SGMA who have been ethnically or racially minoritised reported significantly lower levels of trust in adolescent‐focused researchers, compared to participants from White backgrounds. Importantly, concerns about family rejection are disproportionately experienced, such that SGMA of colour have reported the need to conceal important aspects of their identity (e.g. their sexuality and gender identity), particularly since the start of the COVID‐19 pandemic (Salerno et al., 2022). Some adolescents may also be structurally disadvantaged and ignored/forgotten in research design or mental health intervention delivery, such as SGMA who are transient because they are homeless (Ormiston, 2022), particularly homeless SGMA of colour (Page, 2017). The issue of specific and varied structural barriers to engaging in research (and also with psychological interventions more broadly) further reinforces the need for adult stakeholders to provide an overview of how to better support SGMA. Additionally, SGMA in rural locations, often due to their physical locale, may be disadvantaged in engaging with research or mental health interventions (Telfer et al., 2018). Adult stakeholders can describe how internalised stigma for SGMA appears to manifest to others. Adults may also recognise stigma experiences that are by their very nature difficult for SGMA to be able to clearly identify, especially at developmentally earlier stages of life (Kanbur, 2020).

The psychosocial environment for adolescents incorporates individual (e.g. identity formation and development), interpersonal (e.g. family), institutional (e.g. school) and community levels which can all include protective and risk factors for the mental health of SGMA (Elliott et al., 2022). In the current study, we explored the perspectives of adult stakeholders about what supports or undermines the mental health of SGMA to promote a better understanding of the psychosocial environments that support their mental health (which we refer to as ‘supportive mental health environments’). We analysed the perceptions of a range of key adult informants across familial, educational, health care and legal contexts. In line with past work, these included parents (Bull & D'Arrigo‐Patrick, 2018), professionals based in schools (Harris et al., 2021; Johnson, 2023), youth workers as well as health and care professionals (Álvarez et al., 2023). Going beyond past work, we also included the commissioners of health services, who ultimately play an important role in whether online or offline forms of mental health support are made available to adolescents. Finally, we also included adult stakeholders from law enforcement, who have a professional role in understanding how criminal victimisation occurs in the lives of SGMA, because we recognise the increasing concerns and threat of hate crime towards SGMA in their day‐to‐day lives. This study is part of a wider funded project that involved developing a novel online intervention to support the mental well‐being of SGMA (Lucassen, Samra, et al., 2022) which will also include an investigation into the views of SGMA themselves, and the iterative development of an appropriate intervention in line with these findings (Brown et al., 2024). The views of SGMA gathered for the project, during a series of focus groups, will be reported elsewhere. The present study aims to explore adult stakeholders' perspectives on what supports or undermines the mental health of SGMA in everyday life to better understand how to foster supportive mental health environments for SGMA.

## METHODS

### Ethics

Ethical approval for this study was obtained from The Open University's Human Research Ethics Committee (reference HREC/4059/Lucassen).

### Participants

For inclusion in this study, participants needed to be living in the United Kingdom and to be one (or more) of the following:

- A community‐based professional (e.g. youth worker or policing professional);
- A professional who commissions public health or health and social care services relevant to the needs of SGMA (e.g. a sexual health commissioner);
- A parent or guardian of SGMA; or
- A health or social care professional with a particular interest in supporting SGMA.

### Recruitment

Adult participants were recruited for this study via organisations that had endorsed this research and had agreed to support its implementation. This included two SGMA centres, two county councils in Southeastern England and a UK‐wide national centre for police and policing research. Commissioners of public health or health and social care services were recruited from the wider professional networks of KB and LW. The parents were also recruited via the professional networks of the authors (e.g. from support groups for LGBTQ+ staff members and a parent group hosted by a university).

### Procedures

The audio‐recorded interviews were undertaken by ML (a White non‐British gay male and queer‐identified academic experienced in youth mental health work). These interviews were all conducted online due to the COVID‐19 pandemic. The interviews were semi‐structured, which is an established method of data collection for framework analysis (Furber, 2010). Written informed consent was obtained from each participant prior to the interview commencing. Interviews began with introductions and reiterating the purpose of the interview. This included highlighting to participants that the focus of the wider work was developing a new online intervention to support the mental well‐being of SGMA within the adolescent age range, predominantly teenagers. A semi‐structured interview guide, developed by all the authors, was used in the interviews. All interviews covered two main topic areas, specifically the issues SGMA face that can undermine their mental health and their suggestions about tips or solutions for SGMA to support their mental health. Whilst we aimed to develop a better understanding of supportive environments for SGMA mental health, we also acknowledged the need to explore problems and challenges (i.e. barriers) that need to be overcome to better support SGMA with appropriately and sensitively designed mental health and public health interventions. This is in line with other work which combines the investigation of challenges and facilitators to better understand the support needs of SGMA (Morgan et al., 2023). Questions were open‐ended, for example, ‘…what are some of the challenges or issues that LGBTQ+ young people, 13 to 19 years old, face today?’ and ‘How do LGBTQ+ youth try to manage the challenges or issues, both successfully and unsuccessfully?’. All participants completed a brief demographic questionnaire at the end of their interviews.

### Data analysis

A framework analysis approach was used to analyse the qualitative data collected in the interviews. This approach has been developed in the UK specifically for applied social research (Ritchie & Spencer, 1994) and is increasingly commonplace in medical and health research settings (Gale et al., 2013). Framework analysis is particularly useful for multidisciplinary work in which the participants may include those in a diverse range of roles, including clinicians, service users, caregivers and lay people (Gale et al., 2013). Ritchie and Spencer (1994) outline that framework analysis can helpfully address research questions that are strategic in nature (rather than primarily focused on descriptive or interpretative goals), such as how services can be improved through new plans, policies or actions. The breadth of participant inclusion allows framework analysis to help provide practical findings to inform health research and service design through the notion that it is a ‘whole paradigm approach’ (Kiernan & Hill, 2018, p. 248). Framework analysis encourages organising, indexing and charting of data in line with key themes or issues that can be identified a priori according to the goals of the study (Ritchie & Spencer, 1994). For the present study, the focus on the barriers and facilitators of supporting SGMA mental health was expected to play a role in producing categories for the final framework. In contrast, approaches that are highly data‐driven such as grounded theory or interpretive phenomenological analysis (Charmaz & Thornberg, 2021; Smith et al., 2013) require greater homogeneity in participant sampling and as such would be inappropriate for the current relatively diverse set of participants.

All transcripts were checked for accuracy against the audio recording and then exported and analysed in NVivo 14 (QSR International). The framework analysis approach entails five steps (Ritchie & Spencer, 1994; Ritchie & Spencer, 2002), which were carried out in the present study. As part of the data familiarisation (step one), RS and ANG read through and examined transcripts. RS is a British Asian heterosexual cisgender female with lived experience of serious mental illness diagnosed during adolescence. ANG is a White non‐British queer female counsellor with experience working in the field of LGBTQ+ mental health. As part of constructing the initial framework (step two), RS and ANG discussed and identified eight broad headings for organising the dataset across the different stakeholders (see Furber, 2010). RS and ANG each indexed eight of the transcripts (step three) in line with the broad headings (for example, ‘young peoples’ responses and stress management’). RS and ANG then divided the dataset into two sets and developed smaller data‐driven open codes (Gale et al., 2013) for the revised framework (Ritchie & Spencer, 2002). All indexed codes were then examined together across the whole dataset by RS and ANG for a working analytical framework. Revisions were discussed by RS and ANG to develop the revised theoretical framework which was applied to the entire dataset. The subsequent charting stage (step four) involved searching for patterns that supported the identification of key themes. In the mapping and interpretation final stage (step five), RS initially organised the key themes identified. The final themes were agreed upon after discussion between RS and ANG and subsequent revision. The themes and exemplar quotes were checked for clarity by ML and any issues were resolved with all authors to provide a final analysis.

## RESULTS

Sixteen interviews were conducted between the 19th of October 2021 and the 20th of January 2022. All but one of the participants were aged 30 years or older. Participants reported a range of genders and over half were LGBTQ+. All the participants were British, with 14 being White, one Black and one South Asian (see Table 1 below). The interviews ranged in length from 41 min to 1 h and 11 minutes (mean = 56 min).

**TABLE 1:** Adult expert interviews’ demographics.

Total number of interviews	16 individual interviews	
Type of expert	Community‐based professional (e.g. youth worker)	4 (25.0%)
	Professional who commissions health and social care services	4 (25.0%)
	Parent or guardian of SGMA	4 (25.0%)
	Health or social care professional	4 (25.0%)
Age group	30 years or older	15 (93.8%)
	26–29 years old	1 (6.3%)
Gender (open text responses) to ‘Your gender/gender identity’	Male	6 (37.5%)
	Female	5 (31.3%)
	Cis female	2 (12.5%)
	Cis male	1 (6.3%)
	Male/non‐binary	1 (6.3%)
	Non‐binary	1 (6.3%)
LGBTQ+ identified		9 (56.3%)
Ethnicity (open text responses) to ‘Your ethnic group (e.g. Black British)’	White British	13 (81.3%)
	White Welsh	1 (6.3%)
	Black British	1 (6.3%)
	British/South East Asian	1 (6.3%)

## FINDINGS FROM THE FRAMEWORK ANALYSIS

**FIGURE 1:**
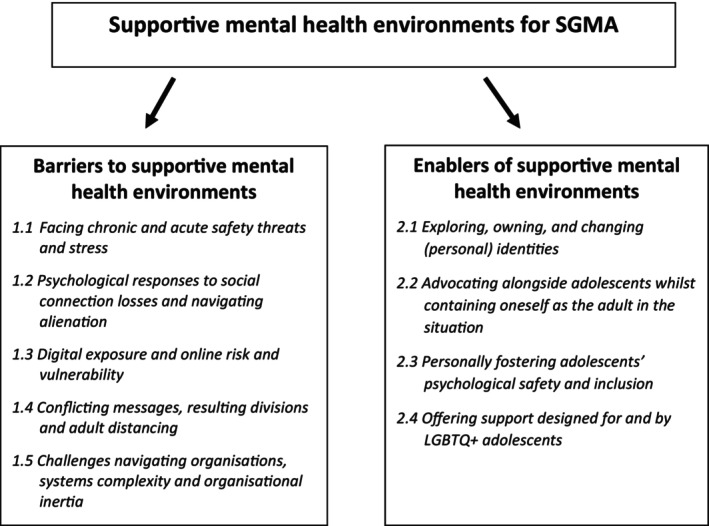
Overview of themes.

The framework analysis resulted in five themes relating to the problems and challenges in the everyday psychosocial environments of SGMA, which have negative consequences for their mental health, entitled ‘Barriers to supportive mental health environments’. Additionally, the framework analysis resulted in four themes that related to the promotion of positive mental health in the everyday psychosocial environments of SGMA, entitled ‘Enablers of supportive mental health environments’, which are outlined below in Figure 1. Excerpts from the interview transcripts are used to illustrate the themes.

### Barriers to supportive mental health environments

#### Facing chronic and acute safety threats and stress

The challenges, stresses and abuses involved in minoritisation in certain cis‐heteronormative cultures (i.e. where being cisgender and heterosexual is normal and always preferred) were described. Participants outlined how SGMA, despite being minors, had to manage their own physical and psychological safety. Some participants commented on how SGMA can sometimes be blamed for the abuse they face and the consequences for the perpetrators should they speak up (‘The kids are always quite scared that it then becomes a cycle of like ‘oh you grassed up my mates. Now they've been suspended just because you're gay. That's not our fault you're gay’…’, P8, Health and social care professional).

The need to manage a range of mistreatment was mentioned including the incorrect uses of their preferred name, pronouns and gender identity as well as being subjected to peer bullying, physical harassment and hate crimes. The normalisation of identity‐based abuse and harassment (particularly homophobia, biphobia and transphobia) present threats to SGMA sense of self and identity and could occur anywhere (e.g. examples experienced on public transport and school were described). Participants also described SGMA being exposed to unwanted and early sexualisation (e.g. crude questions about particular sexual acts) or having to explain or justify their identity compared to their non‐SGMA peers. At school, trans students can face discrimination when using toilets, and a participant noted that trans students sometimes try to avoid urinating during school hours by not drinking which puts them at risk of dehydration. Adults' discomfort or ignorance about these issues meant that SGMA could feel unprotected by people in authority (e.g. the police and their teachers were mentioned as examples) which was outlined by a parent participant:

‘There's teachers now, lecturers now that are gender critical… they will use his pronouns, but they have told him that they don't believe in trans people and things like that. They're quite overtly negative’ (P9, Parent).

Due to the risks of being outed (and the consequences of this in relation to family, peer, teacher, or general social rejection), SGMA often manage by developing multiple selves for different facets of their lives. Some participants also identified that it often becomes the young person's responsibility to identify safe allies and find appropriate sources of support. Unfortunately, the risk of rejection can come with severe safety threats, in particular in terms of negative responses from cultural and religious communities (e.g. a participant described honour‐based violence or abuse and forcing ‘conversion’ or using religious camps as an ‘intervention’). SGMA faced being disowned or being made homeless or being sent to other schools or communities as a result of their sexuality and/or gender identity:

‘A girl came out as being gay and she ended up being intercepted at the airport because they were trying to take her back to Pakistan. They were going to find an arranged marriage for her in Pakistan… it was only because she had managed to approach a police officer at the airport that she was intercepted before, frankly, she disappeared’ (P1, Community‐based professional).

#### Psychological responses to social connection losses and navigating alienation

Many participants described SGMA as facing significant social alienation and relationship losses relating to their sexuality or gender identity. This theme detailed how they psychologically and socially respond to the stigma, mistreatment and abuse they experience and how they navigate othering and social exclusion. Participants described how young people were having to cope with or grieve a loss of connection or group membership to their peers during important stages of their identity development. The gatekeeping of gendered groups and spaces, or being denied care and treatment, was described as encouraging the idea that the young person's existence is ‘wrong’ in some way. A few participants linked this to shame, internalised stigma, self‐hatred or struggling to accept aspects of themselves. This could also include body‐based alienation, particularly during puberty‐related body changes, for those whose bodies do not align with their gender identity. Some participants described how young people might cope by trying to assimilate, conform or use avoidance‐based strategies to ‘fit in’. For example: ‘He [trans son] loved being the goalkeeper [playing football] because it was such a pivotal role. And he just loved the whole camaraderie of it. And since he's come out, he's not played one bit of sport. Partly because he has to bind [i.e. flatten his chest], you know, he has to use a binder and then that limits his breathing. And that's something he really misses’ (P9, Parent).

A few participants noted that SGMA are forced to grow up early or may feel world‐weary at a younger age. There was sometimes also a pressure to conform to LGBTQ+ stereotypes and young people could still face rejection and negativity within the wider LGBTQ+ communities, particularly for bisexuality which could be framed as ‘not making a choice or sitting on the fence’ (P5, Health and social care professional).

#### Digital exposure and online risk and vulnerability

Many participants noted that SGMA frequently looked for online solidarity and communities because they tended to be rejected from aspects of their offline social worlds, but that they may risk being exposed to harm when they go online. Participants also described how young people tend to be naïve or make mistakes online which in turn potentially exposes them to online blackmail or risks to their personal security/safety:

‘…perhaps making unsafe choices especially for young men, trying to get themselves validated [via sexual experiences] in very unsafe ways when they're vulnerable’ (P8, Health and social care professional).

Some participants commented on the ways in which the young person becomes (or fails to become) responsible for managing their own distress or vulnerability online. In describing online safety, some participants explained that it became the young person's responsibility to curate their experiences, by seeking out positive messages and avoiding negativity or engagement in ‘digital self‐harm’ (P1, Community‐based professional), such as reading hateful or abusive content about LGBTQ+ people. A few participants described how some SGMA ‘actively seek out the negativity… because for them they use it as kind of reinforcement for what they believe about themselves’ (P1, Community‐based professional). Another participant described this occurring because there is a belief that there is ‘…something about their identity that's shameful or different or deserves to be harassed or punished’ (P5, Health or social care professional).

#### Conflicting messages, resulting divisions and adult distancing

Participants described confusing or conflicting environments that young people in general have to deal with, and this inadvertently included the participant's own confused messaging (of which they appeared unaware). One participant described ‘myth‐building’ (P2, Community‐based professional), such as creating environments of fear amongst SGMA that left them afraid of particular spaces (e.g. public transport). This participant explained that hate crime statistics did not support the idea that railways were dangerous, whilst simultaneously acknowledging that hate crimes tend to go unreported. It was mentioned that sometimes adults in SGMA environments may inadvertently send messages, perceived as confusing by SGMA, about the adult's own personal belief systems and perceptions of LGBTQ+ people. Such situations can serve to distance them from SGMA. An example was given of the case of a therapist wearing a crucifix (i.e. a religious symbol when Christianity can be associated with LGBTQ+ mistreatment) in sessions which created a potentially confusing situation for SGMA when accessing psychological services. Another conflicting message was how SGMA should handle bullying, which contrasted markedly with the more established approaches (e.g. tell a teacher), such as the idea that the victim should irritate the bully (‘I always say if the bully doesn't like something you do, do it a hundred times more, keep doing it! And it'll really irritate them’ P3, Community‐based professional). Another example of a confusing and conflicting message was inherent in a participant's account that ‘I genuinely believe there's no better time to be LGBT. I mean, I think it will just keep improving, too’ (P1, Community‐based professional) and then from the same participant: ‘I'm so glad I didn't grow up in this generation. To be fair, I don't know how I would have coped’ (P1, Community‐based professional). Importantly, many participants identified conflicting and confusing messaging within SGMA social environments, such as the discrepancy between a school environment perceived as supportive by adults (e.g. teachers) and the actual school experience:

‘If the school's got things like about LGBTQ identities, but if you go into the playground they're still saying boys and girls or not having a gender neutral toilet and a trans kid in the school who is expected to go and use a toilet that says disabled’ (P8, Health or social care professional)

This theme also included conflicts in the participants' accounts which indicated examples of adult distancing from these issues. For example, some participants described generalisations about young people who are ‘are quite notorious for not understanding things very well’ (P4, Community‐based professional) and SGMA, including their unworldliness, short attention spans, and how they change their behaviours and ideas rapidly, which stood in contrast to other messaging around how SGMA are forced to mature earlier.

#### Challenges navigating organisations, systems complexity and organisational inertia

Participants who were professionals described how difficult or complicated it was to take a proactive stance on supporting the mental health of SGMA. They detailed many reasons that underlie a lack of progress in this area, often related to the complexity of their organisational systems. Some key issues described related to the bureaucracy and politics of the procurement processes and the complicated funding landscape in which there are changing targets, such that there may be no key performance indicators or metrics for SGMA specifically (‘…if there is a public health outcome next to it then if we can measure it, we'll measure it. I don't think there are any outcomes for LGBT youth’ P12, Commissioner). There appeared to be some confusion about whether the mental well‐being of SGMA related mainly to state‐funded National Health Service mental health services per se or also the ‘sexual health contract’ (P13, Commissioner), which is linked to supporting the provision of Personal, Social, Health and Economic (PSHE) education in schools in England. Alternatively, a public health professional questioned whether SGMA mental health was an ‘education issue or health issue’ (P12, Commissioner), meaning that it was unclear whether funding for improvements and services should come from health care or education authorities. It was suggested that organisational inertia was underscored by the problem of ‘where does it sit?’ (P12, Commissioner). Importantly, there was also a failure to develop ongoing intelligence regarding the issue of SGMA and their health issues:

‘I honestly don't know… we sadly lack a lot of understanding of how our services are either helping or hindering or what our services are. I wouldn't even be able to tell you how many young people or adults there are that identify as transgender… We've changed and with each contract we renew, we change our demographic requirements to reporting’ (P14, Commissioner).

In thinking about the challenges of commissioning specific services, there was concern that commissioners may not feel confident in choosing optimal services for young people, ‘…we haven't just bought some really ropey Myspace [i.e. an obsolete platform] kind of thing, because what the hell do we know, we're all adults. Have we bought something that's credible for a 14/15‐year‐old? And there's always a bit of a wing and a prayer with that one’ *(*P12, Commissioner). There was also mention of concerns about facing a ‘backlash’ (P6, Health or social care professional), particularly from faith‐based groups when offering sex education that is inclusive for SGMA. Organisational inertia about supporting SGMA mental health also extended to schools who ‘will always say they don't have any money’ (P12, Commissioner) and were often organisational siloes: ‘Each school is a sovereign and doesn't need to speak to anyone else’ (P12, Commissioner).

### Enablers of supportive mental health environments

#### Exploring, owning and changing (personal) identities

Commonly, participants described how young people actively explore and develop their own evolving personal identities to better understand themselves and their challenges, and in doing so they learn to cope and feel better in themselves. According to a number of participants, SGMA might research and look for guidance online relating to their gender identity, gender expression and sexuality and may also research how to help others in their lives understand their journey and identity development:

‘…he [trans son] obviously found something about how you get your parents to become part of the process about choosing your new name… he wrote me two notes, so he wrote me first when he came out as gay and then as transgender… I'd never say this to him, but they were slightly formulaic, I could tell almost like there were some sentences that weren't him, that he'd obviously copied down and had inserted bits’ (P9, Parent).

Some participants reported that aspects of this learning and developmental journey seemed therapeutic, such as when SGMA feel able to express themselves through their appearance and present authentically as themselves whilst they grow and evolve. SGMA also look to connect with others on a similar journey and stage of that journey (rather than simply with other adolescents of a comparable age). Some participants described how SGMA being trusted to explore their identities and connect with others allowed them to learn about themselves (whether through offline interactions or their online digital lives). Importantly, SGMA interacting positively with empathic people, finding role models or social visibility through these methods (e.g. at pride events, via school peers, faith‐based communities and social media influencers) seemed to validate difference and fostered belonging to a community.

#### Advocating alongside adolescents whilst containing oneself as the adult in the situation

Many participants described how adults can advocate for SGMA. For instance, it was mentioned that adults can highlight the young person's rights along their journey, and signpost and connect them to appropriate help, support and care. However, as described by parent participants, adults need to carefully balance understanding the young person's anger and disappointment at times whilst also trying to stay hopeful and positive when necessary. There might be a careful balancing act needed in terms of SGMA expectations and between hope and reality, for example, the risks as well as the benefits of transitioning for trans adolescents which was mentioned by a parent participant: ‘…you [i.e. SGMA] can start this journey if you want to, but it's a very long and painful one and you might get to the end of it and be still unfulfilled’ P11, Parent). It was described as important to control and manage one's own beliefs, values and feelings (as an adult) to support SGMA effectively. It was mentioned that parents need to be mindful of their own mental health and get support when needed. Some parent participants described how they had to contain their own emotions (such as fear, worry, concern, anger and outrage) and manage their own defensive reactions:

‘I suppose he wants to handle it and I suppose it's the same with any teenager, wanting your parents to be sympathetic without them going off on one… So, also saying, do you want us to do this, do you want us to help by contacting the school? And of course it's always, no, he never does want us to do that’ (P9, Parent).

For those working in organisational contexts, participants described how their role involves bridging the divide between ‘them’ and ‘us’ and creating a better culture of tolerance and acceptance of differences. Many participants spoke about the need for continual education or self‐directed learning to understand and navigate the evolving language and terminology that SGMA use so adults can sensitively support them. This involves learning from one's mistakes, apologising, and teaching oneself important aspects from the experiences of people who are LGBTQ+. Some professional participants described how the fear of saying the wrong thing affected their behaviour and that of others within their organisation. There was a sense that asking LGBTQ+ people to help inform service design and educate LGBTQ+ allies about anti‐oppressive practices helped promote better organisational action.

#### Personally fostering adolescents' psychological safety and inclusion

Participants described the day‐to‐day practices and behaviours that adults can do to allow the young person to feel safe and secure to share and be open in their own time. This involved engaging and showing interest in what the young person enjoys (‘That's one of the things we do, we sit there as a family and watch RuPaul [i.e. RuPaul's Drag Race, an LGBTQ+ television series]’ P9, Parent). This involves sometimes not asking direct questions and at times talking whilst doing other activities, so that the communication feels less intense and pressured. Some practitioner professionals explained how it was important to be aware of what the young person sees as acceptable in terms of their boundaries and what stresses them or makes them uncomfortable. For example, SGMA may not want to be acknowledged in public spaces as it could result in ‘outing’ them: ‘… quite often I'll be in a school and there'll be a kid that I might have worked with for a whole year but if I see them in the corridor we just ignore each other’ P8, Health or social care professional). When professionals can engage with SGMA, it was considered important to ‘…just be more mindful… everyone's different, and to use the appropriate name, appropriate pronoun, to be respectful of people's appearances or how they choose to appear and identify’ (P6, Health or social care professional).

Modelling patience and open‐mindedness in interpersonal encounters and trying not to be judgemental were commonly mentioned by various participants. This included being accepting of confusing feelings and being aware that it's acceptable to not know all the answers. As SGMA are at risk of feelings of shame and internalised stigma, adults should embrace and celebrate the young person's individuality. Reminding them about self‐care and looking after themselves can help ‘build them up’ because for some young people to ‘love themselves is a big struggle’ (P7, Health or social care professional).

#### Offering support designed for and by LGBTQ+ adolescents

Participants described what would work or what was needed in developing online interventions for SGMA to improve their mental health. It is important to use the young persons' terminology and create powerful and engaging lasting messages (‘Is it dry, is it dull, is it boring? Just to speak their language, you know’ P2, Community‐based professional). Online resources should use colourful and welcoming visuals that are extensive and resources should not be overly ‘wordy’ (P5, Health or social care professional). It was mentioned that SGMA can be very active and self‐directed learners and so interventions should reflect their desire for discovery, rather than attempt to get potential users to assimilate large amounts of information. For SGMA who have been disappointed by institutional structures (e.g. their school, or governmental and health services), the resource should come from someone they trust that ‘can't be too establishment’ (P9, Parent). Some participants mentioned the need for diverse coming out stories or experiences (such as contributions from those with different faith and cultural backgrounds), as well as exploring the decision to not come out. Participants' ideas about content included advice about how to navigate relationships and talk to other people about your identity. SGMA were identified as needing content around support with conflicted feelings, managing intense feelings and unhelpful thoughts and how to address the shame that can come from marginalisation. Psychological exercises to help SGMA manage their stress levels and promote relaxation were also mentioned. Adults supporting SGMA coming out experiences were perceived as an important and ongoing process:

‘You're going to have to be prepared to come out numerous times. So it's about kind of like giving young people the tools: self‐resilience, self‐acceptance, self‐worth, building up their self‐esteem…’ (P7, Health or social care professional).

The importance of confidentiality and discretion in using online resources was mentioned, such as an emergency shutdown function to hide the intervention from others if needed. Safety strategies and links to other websites and sources were described as ways SGMA might be able to safely connect to other sources of support. Some participants mentioned the value of celebrating LGBTQ+ pioneers and history, and that awareness raising might support the adolescent user's activism in relation to social issues.

## DISCUSSION

There has been considerable research on the problems or challenges SGMA face but there is limited published work in the peer‐reviewed literature focused on interventions or feasible solutions for SGMA. A scoping review of the literature identified only 17 unique interventions internationally which are focused on improving SGMA mental health (Lucassen, Núñez‐García, et al., 2022) which indicates a global research lag disproportionate to the urgency of the issues. The current findings about adult stakeholders' perspectives on identifying the barriers and facilitators of SGMA mental health can usefully inform intervention design and promote supportive practices from adults.

### Supporting adolescents' developing identities offline and online

The findings call for the need to create resources that support the young person's life journey and help SGMA develop their own unique sense of identity whilst acknowledging that these processes are not necessarily temporally bound by traditional notions of ages and stages of identity formation. In line with Halberstam's (2003) work on queer temporalities, the present study demonstrated how SGMA can be at different stages of their journey and seek to situate themselves with like‐minded peers in terms of their journey rather than peers matching their chronological age. We urge that digital interventions and resources for SGMA reflect queer temporalities and not assume that a young person is, or is not, at any particular development stage related to their gender and/or sexuality based solely on their age in years. Our exploratory findings indicate the need to raise awareness about digital risks and highlight protective strategies to assist SGMA in determining who they can trust, both in their online and offline environments, to support personal safety along their self‐development journeys. Interestingly participants reported how SGMA sometimes like to involve supportive adults in their journeys but that this required the adult to appreciate that it is a continual self‐learning process for them as well. Therefore, digital resources and other supports for SGMA could also be used by adults to promote their own learning and self‐development, or as a tool for reflection and discussion amongst their adult peers.

The findings indicate that SGMA can face distressing behaviour from their peers, particularly in school settings, and this by extension involves adults who are tasked with looking after them. For example, teachers can express gender‐critical views in a manner that SGMA can find upsetting, such as deliberately using an incorrect name and pronouns, which poses a problem for SGMA in obtaining safe adult allies. Alternatively, teachers might not know how to react to trans‐, bi‐ or homo‐phobic bullying or fail to identify abuse in the classroom. Instead, they may treat this as merely another disruptive classroom behaviour, rather than identify the harms of bullying related to gender or sexuality directly. Past work has highlighted how school‐based professionals might not know how to address abuse related to sexuality or gender identity (Zeeman et al., 2017) but our findings suggested that there are additional knock‐on effects of SGMA. For example, disappointments related to adults in one environment (e.g. school) can engender feelings of hopelessness in other domains (e.g. psychological therapies) and interfere with SGMA help‐seeking, which adults should be made aware of. We posit that there is a need to generate adult‐focused educational resources on identifying and acting appropriately in school settings (Department for Education, 2023) which makes clearer the interconnectedness of the young person's psychosocial environment.

### Social inclusion and exclusion

The issue of hostility and intolerance at schools has resulted in some gender minoritised adolescents avoiding school toilets, which has an impact on their general health. This is a pertinent issue as it aligns with wider societal discourse regarding judgements about which bodies are suitable for which spaces, with a particular focus on cisgender washing and toilet facilities (Ingrey, 2018). There is a growing awareness that issues relating to body autonomy link transphobia, transmisogyny and (dis)ablism (Slater & Liddiard, 2018). Our findings call for further investigation into the construction of these discriminatory attitudes and practices being demonstrated in the school environment by adults and peers. The current findings reinforce Ingrey's (2018) problematisation of the cisgendering of school toilets and demonstrate how social and political discourse (often led by adults in the media) can play out particularly within the day‐to‐day lives of gender minoritised adolescents by affecting the physical territory that they feel able to use or occupy. Future research could explore the extent to which school‐based social exclusions prompt the often‐noted pursuit of digital spaces and the need for community amongst SGMA. SGMA experiencing greater levels of social exclusion might indicate struggling via digital activities, including their social media use, which could, in turn, help an adult recognise their challenging experiences and thus support them accordingly.

In home settings, the findings highlighted how SGMA parents' belief systems can underpin forced social exclusion from SGMA desired activities and social groups, such as banning LGBTQ+‐related after‐school activities. There exist complex issues relating to the disproportionality of threats and harm for SGMA from particular religious or cultural communities, such as the threat of being sent abroad and other ‘conversion’ attempts. This disproportionality is in line with past work, for example, in Blosnich et al.'s (2020) study on sexually minoritised adults in the USA who had experienced sexual orientation change efforts (i.e. conversion attempts), 80% identified that this was conducted by a religious leader. There is potential for future research avenues to explore how to develop culturally sensitive education about how to identify harmful practices relating to sexual orientation and gender change efforts, without stereotyping or further marginalising particular religious and cultural communities.

On a positive note, efforts to promote social inclusion were mentioned by many participants as key facilitators of positive mental health for SGMA. These included identifying role models (particularly online or in the media), seeing examples of LGBTQ+ social visibility as beneficial, learning about diverse coming out experiences (and conversely, stories of not coming out) and community‐related celebration efforts such as pride or LGBTQ+ history events.

### Building SGMA trust

The need to carefully develop psychological safety and establish trust with SGMA is an important finding from this study that is pertinent to psychotherapeutic and psychological practitioners but also to intervention design. The findings demonstrated the particular importance of digital and online environments to SGMA, which reinforces past work linking offline social exclusion and SGMA mistreatment can drive them to find communities online (Lucassen et al., 2018). The current findings also point to the need for digital support to avoid being seen as too ‘mainstream’ or establishment. Critically, SGMA frequently locate their mistreatment and abuse within wider societal attitudes and institutional structures (including schools and health services). Therefore, support and interventions may need to be designed cognisant of the possibility that organisations from within these structures may not engender the same level of trust and respect amongst SGMA as their non‐SGMA peers. Additionally, individuals working within these structures may need to work to build trust with SGMA.

### Structural issues

Across participants, it was generally described that SGMA face specific threats to their mental health relating to issues around gender and sexuality, in line with minority stress theory (Meyer, 2003). However, a number of participants described inertia or barriers within wider organisations in promoting positive change. This is consistent with the increasing focus on research investigating how the broader structural contexts in which LGBTQ+ populations are living shape their health (Hatzenbuehler et al., 2024). For example, commissioners highlighted a lack of monitoring of the well‐being of SGMA populations which meant they were very unaware of their needs at a local population level. The importance of data monitoring in commissioning contexts is reinforced by Jager et al.'s (2023) realist review of 92 studies on the usage of data in NHS primary care. This review outlined that commissioners consider data on health inequalities as particularly useful to making commissioning decisions and allow commissioners to ‘drill down’ (p. 5) on segments of a population to understand service use and make targeted commissioning improvements. The invisibility of SGMA populations through a lack of data collection or data sharing presents a significant barrier to responding to SGMA mental health needs. This then further hinders action, even when this is encouraged in policy documents, including the UK government's ‘LGBT Action Plan’ (Government Equalities Office, 2018) which actively endorses efforts to improve mental health care for LGBTQ+ individuals. With this in mind, tools developed for improving SGMA mental health may need to encourage organisations to collect appropriate and useful data for assessing the well‐being of SGMA populations to address the monitoring gap and promote targeted commissioning decisions and improvements (e.g. Jager et al., 2023).

Our findings indicated it was unclear whether SGMA mental health is within the remit of education or health. In line with this, the Government in England has been criticised for not setting out the actions or budget for delivering on its mental health strategy for children and adolescents, ‘Future in Mind’, which has resulted in insufficient cross‐departmental co‐operation (Garratt et al., 2024). Therefore, the findings presented here may represent the lack of clarity regarding governmental departmental responsibility for all adolescents' mental health, rather than SGMA specifically. However, in relation to SGMA, contemporary governmental guidance on promoting young people's mental health in schools can still fail to outline differential or particular risks for SGMA (e.g. Office for Health Improvement and Disparities & Department for Education, 2023) which we consider a missed opportunity for sharing important knowledge about mental health risks. In our study, schools were described as siloes, and, resultingly, good practice was not identified or shared across schools. This is consistent with past work which recommends funding and support need to be developed beyond the individual school level to address adolescent mental health in these settings (O'Reilly et al., 2018).

In this study, participants highlighted that using LGBTQ+ allies to teach about anti‐oppressive practices could be helpful in changing everyday environments (for example, in education, in the police, in health care or in schools). This is in line with Jessiman et al.'s (2022) participative action research study in three UK secondary schools which recommended embedding LGBTQ+ role models across all subject areas, and adopting an inclusive curriculum representing the diversity of the student body. They reported that these inclusive practices foster a more supportive school culture for the mental health of minoritised students, particularly SGMA in this context, as well as the wider student group.

### Strengths

A strength of this work is the use of a range of key adult stakeholders who shared their views about the mental well‐being of SGMA, for example, the sample included policing and health commissioning professionals. The decision to include commissioners was novel to this study and highlighted key structural barriers such as the unresolved conflicts about the funding remit and responsibility for SGMA mental health. Addressing this issue would require more explicit policy leadership with requirements on public health, mental health services and education providers to collaborate more effectively, for example, by requiring the collection of relevant SGMA demographics and outcomes. This paper could contribute to the discussions about equality monitoring data for adolescent‐focused services.

The findings help in identifying what experiences might look like (to adults) for some SGMA who are traditionally and systematically under‐served. In particular, SGMA are disadvantaged by their exclusion from research and who are also poorly served in terms of mental health support. For example, participants spoke about the different ways that SGMA not routinely involved in research (e.g. those who are questioning or unsure of their identities) seek to address their issues more privately (e.g. searching for answers online or asking questions on platforms like Reddit). This highlights the challenge of involving young people who are questioning or unsure about their sexuality and/or gender in research but also underscores the clear need to make efforts for their inclusion. The present work was inclusive across the spectrum of diverse sexual and gender categories of SGMA which is also consistent with research identifying that adolescents can resist or oppose traditional labelling and categories (Hammack et al., 2022).

### Limitations

There are some key study limitations. The sample of 16 individuals is small and is not designed to be representative of the wider adult population (for instance, over half of the adult stakeholders were LGBTQ+). The study was situated primarily in England and the findings may not capture structural or cultural factors pertinent to other UK nations or outside the UK. Due to the focus on particular locales and organisations, the sample population and their views may be skewed to reflect issues and experiences within these specific areas. It is also extremely likely that parents who participated in this study represent those who have proactively attempted to support their child, compared to parents who have ongoing issues related to the acceptance of their child's sexuality and gender. As the present work was a small‐scale exploratory study into the views of adult stakeholders, the implications and conclusions from this work should be treated with caution due to the somewhat limited respondent group. Further corroboration of findings is needed. In future studies, we recommend sampling a greater number of participants drawn from a wider range of geographical locations to better represent adults in the range of professional and personal roles included in the present work.

### Conclusion

Professionals and parents identified barriers as well as enablers related to creating supportive mental health environments for SGMA. Understandably, challenging social environments affect SGMA's psychological safety as well as their trust in adults, and this in turn can lead to issues related to SGMA help‐seeking. Adults aiming to be supportive of SGMA should become aware of the pertinent stressors related to their minoritisation experiences to better understand the young person. Recognising and addressing these specific stressors for SGMA can enable supportive mental health environments.

## AUTHOR CONTRIBUTIONS

**Rajvinder Samra:** Methodology; funding acquisition; formal analysis; writing – review and editing; writing – original draft; investigation. **Mathijs F. G. Lucassen:** Conceptualization; funding acquisition; project administration; data curation; writing – review and editing. **Alicia Núñez‐García:** Formal analysis; project administration; software; data curation. **Katherine E. Brown:** Writing – review and editing; funding acquisition. **Katharine A. Rimes:** Writing – review and editing; funding acquisition. **Louise M. Wallace:** Funding acquisition; writing – review and editing.

## FUNDING INFORMATION

The funding for this project was provided by the UK Medical Research Council (grant reference MR/V031449/1).

## CONFLICT OF INTEREST STATEMENT

All authors declare that they have no conflicts of interest.

## Data Availability

This qualitative study includes in‐depth interviews covering sensitive topics. Although identifying information was removed in this paper, the full anonymised transcripts alongside participant demographics may be identifiable to people who know the participants. Therefore, there are ethical and privacy concerns relating to data sharing. However, data requests may be submitted to rajvinder.samra@open.ac.uk for consideration in consultation with the broader study team.

## References

[papt12548-bib-0001] Álvarez, R. G. , Hofman, S. , ten Brummelaar, M. , & López, M. L. (2023). Care professionals' perspectives and roles on resilience among LGBTQIA+ youth in out‐of‐home care: A multidimensional perspective. Children and Youth Services Review, 150, 107012. 10.1016/j.childyouth.2023.107012

[papt12548-bib-0002] Blosnich, J. R. , Henderson, E. R. , Coulter, R. W. , Goldbach, J. T. , & Meyer, I. H. (2020). Sexual orientation change efforts, adverse childhood experiences, and suicide ideation and attempt among sexual minority adults, United States, 2016–2018. American Journal of Public Health, 110(7), 1024–1030. 10.2105/AJPH.2020.305637 PMC7287530 32437277

[papt12548-bib-0003] Brown, K. , Lucassen, M. F. , Núñez‐García, A. , Rimes, K. A. , Wallace, L. M. , & Samra, R. (2024). A web‐based intervention to support the mental well‐being of sexual and gender minority young people: Mixed methods co‐design of oneself. JMIR Formative Research, 8, e54586. 10.2196/54586 38772025 PMC11150889

[papt12548-bib-0004] Bull, B. , & D'Arrigo‐Patrick, J. (2018). Parent experiences of a child's social transition: Moving beyond the loss narrative. Journal of Feminist Family Therapy, 30(3), 170–190. 10.1080/08952833.2018.1448965

[papt12548-bib-0005] Charmaraman, L. , Hodes, R. , & Richer, A. M. (2021). Young sexual minority adolescent experiences of self‐expression and isolation on social media: Cross‐sectional survey study. JMIR Mental Health, 8(9), e26207. 10.2196/26207 34524107 PMC8482247

[papt12548-bib-0006] Charmaz, K. , & Thornberg, R. (2021). The pursuit of quality in grounded theory. Qualitative Research in Psychology, 18(3), 305–327. 10.1080/14780887.2020.1780357

[papt12548-bib-0007] Cooper, R. M. , & Blumenfeld, W. J. (2012). Responses to cyberbullying: A descriptive analysis of the frequency of and impact on LGBT and allied youth. Journal of LGBT Youth, 9(2), 153–177. 10.1080/19361653.2011.649616

[papt12548-bib-0008] Day, J. K. , Perez‐Brumer, A. , & Russell, S. T. (2018). Safe schools? Transgender youth's school experiences and perceptions of school climate. Journal of Youth and Adolescence, 47(8), 1731–1742. 10.1007/s10964-018-0866-x 29858740 PMC7153781

[papt12548-bib-0009] Department for Education . (2023). Gender questioning children: Non‐statutory guidance for schools and colleges in England. Retrieved from https://consult.education.gov.uk/equalities‐political‐impartiality‐anti‐bullying‐team/gender‐questioning‐children‐proposed‐guidance/supporting_documents/Gender%20Questioning%20Children%20%20nonstatutory%20guidance.pdf

[papt12548-bib-0010] Dunlop, B. J. , Hartley, S. , Oladokun, O. , & Taylor, P. J. (2020). Bisexuality and non‐suicidal self‐injury (NSSI): A narrative synthesis of associated variables and a meta‐analysis of risk. Journal of Affective Disorders, 276, 1159–1172. 10.1016/j.jad.2020.07.103 32823255

[papt12548-bib-0011] Elliott, K. J. , Stacciarini, J. M. R. , Jimenez, I. A. , Rangel, A. P. , & Fanfan, D. (2022). A review of psychosocial protective and risk factors for the mental well‐being of rural LGBTQ+ adolescents. Youth & Society, 54(2), 312–341. 10.1177/0044118X211035944

[papt12548-bib-0012] Fenaughty, J. , Lucassen, M. F. G. , Clark, T. , & Denny, S. (2019). Factors associated with academic achievement for sexual and gender minority and heterosexual cisgender students: Implications from a nationally representative study. Journal of Youth and Adolescence, 48, 1883–1898. 10.1007/s10964-019-01124-w 31520237

[papt12548-bib-0013] Furber, C. (2010). Framework analysis: A method for analysing qualitative data. African Journal of Midwifery and Women's Health, 4(2), 97–100. 10.12968/ajmw.2010.4.2.47612

[papt12548-bib-0014] Gale, N. K. , Heath, G. , Cameron, E. , Rashid, S. , & Redwood, S. (2013). Using the framework method for the analysis of qualitative data in multi‐disciplinary health research. BMC Medical Research Methodology, 13(1), 117. 10.1186/1471-2288-13-117 24047204 PMC3848812

[papt12548-bib-0015] Garratt, K. , Kirk‐Wade, E. , & Long, R. (2024). Children and young people's mental health: Policy and services (England). Commons Library Research Briefing (CBP‐7196). Retrieved from. https://researchbriefings.files.parliament.uk/documents/CBP‐7196/CBP‐7196.pdf

[papt12548-bib-0016] Government Equalities Office . (2018). LGBT Action Plan 2018: Improving the lives of lesbian, gay, bisexual and transgender people. Retrieved from https://www.gov.uk/government/publications/lgbt‐action‐plan‐2018‐improving‐the‐lives‐of‐lesbian‐gay‐bisexual‐and‐transgender‐people/lgbt‐action‐plan‐2018‐improving‐the‐lives‐of‐lesbian‐gay‐bisexual‐and‐transgender‐people

[papt12548-bib-0017] Halberstam, J. (2003). What's that smell?: Queer temporalities and subcultural lives. International Journal of Cultural Studies, 6(3), 313–333. 10.1177/13678779030063005

[papt12548-bib-0018] Hammack, P. L. , Hughes, S. D. , Atwood, J. M. , Cohen, E. M. , & Clark, R. C. (2022). Gender and sexual identity in adolescence: A mixed‐methods study of labeling in diverse community settings. Journal of Adolescent Research, 37(2), 167–220. 10.1177/07435584211000315

[papt12548-bib-0019] Harris, R. , Wilson‐Daily, A. E. , & Fuller, G. (2021). Exploring the secondary school experience of LGBT+ youth: An examination of school culture and school climate as understood by teachers and experienced by LGBT+ students. Intercultural Education, 32(4), 368–385. 10.1080/14675986.2021.1889987

[papt12548-bib-0020] Hatzenbuehler, M. L. , Lattanner, M. R. , McKetta, S. , & Pachankis, J. E. (2024). Structural stigma and LGBTQ+ health: A narrative review of quantitative studies. The Lancet Public Health, 9(2), e109–e127. 10.1016/S2468-2667(23)00312-2 38307678

[papt12548-bib-0021] Ingrey, J. (2018). Problematizing the cisgendering of school washroom space: Interrogating the politics of recognition of transgender and gender non‐conforming youth. Gender and Education, 30(6), 774–789. 10.1080/09540253.2018.1483492

[papt12548-bib-0022] Jager, A. , Wong, G. , Papoutsi, C. , & Roberts, N. (2023). The usage of data in NHS primary care commissioning: A realist review. BMC Medicine, 21(1), 236. 10.1186/s12916-023-02949-w 37400837 PMC10318817

[papt12548-bib-0023] Jessiman, P. , Kidger, J. , Spencer, L. , Geijer‐Simpson, E. , Kaluzeviciute, G. , Burn, A. M. , Leonard, N. , & Limmer, M. (2022). School culture and student mental health: A qualitative study in UK secondary schools. BMC Public Health, 22, 619. 10.1186/s12889-022-13034-x 35351062 PMC8964383

[papt12548-bib-0024] Johnson, B. (2023). Exploring the impact of panoptic heteronormativity on UK primary teachers advocating for LGBTQ+ inclusive education. Education, Citizenship and Social Justice, 19, 202–217. 10.1177/17461979231151615

[papt12548-bib-0025] Kanbur, N. (2020). Internalized homophobia in adolescents: Is it really about culture or religion? Journal of the Canadian Academy of Child and Adolescent Psychiatry, 29(2), 124–126. 32405314 PMC7213920

[papt12548-bib-0026] Kiernan, M. D. , & Hill, M. (2018). Framework analysis: A whole paradigm approach. Qualitative Research Journal, 18(3), 248–261. 10.1108/QRJ-D-17-00008

[papt12548-bib-0027] Liu, R. T. , Sheehan, A. E. , Walsh, R. F. L. , Sanzari, C. M. , Cheek, S. M. , & Hernandez, E. M. (2019). Prevalence and correlates of non‐suicidal self‐injury among lesbian, gay, bisexual, and transgender individuals: A systematic review and metaanalysis. Clinical Psychology Review, 74, 101783. 10.1016/j.cpr.2019.101783 31734440 PMC6896220

[papt12548-bib-0028] Lucassen, M. , Samra, R. , Iacovides, I. , Fleming, T. , Shepherd, M. , Stasiak, K. , & Wallace, L. M. (2018). How LGBT+ young people use the internet in relation to their mental health and envisage the use of e‐therapy: Exploratory study. JMIR Serious Games, 6(4), e11249. 10.2196/11249 30578194 PMC6320432

[papt12548-bib-0029] Lucassen, M. F. G. , Merry, S. N. , Robinson, E. M. , Denny, S. , Clark, T. , Ameratunga, S. , Crengle, S. , & Rossen, F. V. (2011). Sexual attraction, depression, self‐harm, suicidality and help‐seeking behaviour in New Zealand secondary school students. The Australian and New Zealand Journal of Psychiatry, 45(5), 376–383. 10.3109/00048674.2011.559635 21361850

[papt12548-bib-0030] Lucassen, M. F. G. , Núñez‐García, A. , Rimes, K. A. , Wallace, L. M. , Brown, K. E. , & Samra, R. (2022). Coping strategies to enhance the mental wellbeing of sexual and gender minority youths: A scoping review. International Journal of Environmental Research and Public Health, 19(14), 8738. 10.3390/ijerph19148738 35886595 PMC9319075

[papt12548-bib-0031] Lucassen, M. F. G. , Samra, R. , Rimes, K. A. , Brown, K. E. , & Wallace, L. M. (2022). Promoting resilience and well‐being through co‐design (The PRIDE Project): Protocol for the development and preliminary evaluation of a prototype resilience‐based intervention for sexual and gender minority youth. JMIR Research Protocols, 11(2), e31036. 10.2196/31036 35103613 PMC8848231

[papt12548-bib-0032] Lucassen, M. F. G. , Stasiak, K. , Samra, R. , Frampton, C. M. , & Merry, S. N. (2017). Sexual minority youth and depressive symptoms or depressive disorder: A systematic review and meta‐analysis of population‐based studies. The Australian and New Zealand Journal of Psychiatry, 51(8), 774–787. 10.1177/0004867417713664 28565925

[papt12548-bib-0033] Macapagal, K. , Coventry, R. , Arbeit, M. R. , Fisher, C. B. , & Mustanski, B. (2017). “I won't out myself just to do a survey”: Sexual and gender minority adolescents' perspectives on the risks and benefits of sex research. Archives of Sexual Behavior, 46(5), 1393–1409. 10.1007/s10508-016-0784-5 27469352 PMC5274602

[papt12548-bib-0034] Marchi, M. , Arcolin, E. , Fiore, G. , Travascio, A. , Uberti, D. , Amaddeo, F. , Converti, M. , Fiorillo, A. , Mirandola, M. , Pinna, F. , Ventriglio, A. , Galeazzi, G. M. , & Italian Working Group on LGBTIQ Mental Health . (2022). Self‐harm and suicidality among LGBTIQ people: A systematic review and meta‐analysis. International Review of Psychiatry, 34(3–4), 240–256. 10.1080/09540261.2022.2053070 36151841

[papt12548-bib-0035] Meyer, I. H. (2003). Prejudice, social stress, and mental health in lesbian, gay, and bisexual populations: Conceptual issues and research evidence. Psychological Bulletin, 129(5), 674–697. 10.1037/0033-2909.129.5.674 12956539 PMC2072932

[papt12548-bib-0036] Morgan, H. , Wells, L. , Lin, A. , Strauss, P. , & Perry, Y. (2023). Parental challenges, facilitators and needs associated with supporting and accepting their trans child's gender. LGBTQ+ Family: An Interdisciplinary Journal, 19(1), 70–86. 10.1080/27703371.2022.2142717

[papt12548-bib-0037] Moyano, N. , & Sánchez‐Fuentes, M. D. M. (2020). Homophobic bullying at schools: A systematic review of research, prevalence, school‐related predictors and consequences. Aggression and Violent Behavior, 53, 101441. 10.1016/j.avb.2020.101441

[papt12548-bib-0038] Office for Health Improvement and Disparities & Department for Education . (2023). Promoting children and young people's mental health and wellbeing. Retrieved from https://www.gov.uk/government/publications/promoting‐children‐and‐young‐peoples‐emotional‐health‐and‐wellbeing

[papt12548-bib-0039] O'Reilly, M. , Adams, S. , Whiteman, N. , Hughes, J. , Reilly, P. , & Dogra, N. (2018). Whose responsibility is Adolescent's mental health in the UK? Perspectives of key stakeholders. School Mental Health, 10, 450–461. 10.1007/s12310-018-9263-6 30464778 PMC6223973

[papt12548-bib-0040] Ormiston, C. K. (2022). LGBTQ youth homelessness: Why we need to protect our LGBTQ youth. LGBT Health, 9(4), 217–221. 10.1089/lgbt.2021.0324 35325559

[papt12548-bib-0041] Page, M. (2017). Forgotten youth: Homeless LGBT youth of color and the runaway and homeless youth act. Northwestern Journal of Law & Social Policy, 12(2), 17–45. https://scholarlycommons.law.northwestern.edu/njlsp/vol12/iss2/2

[papt12548-bib-0042] Pompili, M. , Lester, D. , Forte, A. , Seretti, M. E. , Erbuto, D. , Lamis, D. A. , Amore, M. , & Girardi, P. (2014). Bisexuality and suicide: A systematic review of the current literature. The Journal of Sexual Medicine, 11(8), 1903–1913. 10.1111/jsm.12581 24839908

[papt12548-bib-0043] Rimes, K. A. , Goodship, N. , Ussher, G. , Baker, D. , & West, E. (2019). Non‐binary and binary transgender youth: Comparison of mental health, self‐harm, suicidality, substance use and victimization experiences. The International Journal of Transgenderism, 20(2–3), 230–240. 10.1080/15532739.2017.1370627 32999609 PMC6831005

[papt12548-bib-0044] Ritchie, J. , & Spencer, L. (1994). Qualitative data analysis for applied policy research. In A. Bryman & R. Burgess (Eds.), Analysing qualitative data (pp. 173–194). Routledge.

[papt12548-bib-0045] Ritchie, J. , & Spencer, L. (Eds.). (2002). Qualitative data analysis for applied policy research. Sage.

[papt12548-bib-0046] Salerno, J. P. , Gattamorta, K. A. , & Williams, N. D. (2022). Impact of family rejection and racism on sexual and gender minority stress among LGBTQ+ young people of colour during COVID‐19. Psychological Trauma Theory Research Practice and Policy, 15(4), 637–647. 10.1037/tra0001254 35511543 PMC10361835

[papt12548-bib-0047] Sawyer, S. M. , Azzopardi, P. S. , Wickremarathne, D. , & Patton, G. C. (2018). The age of adolescence. The Lancet Child & Adolescent Health, 2(3), 223–228. 10.1016/S2352-4642(18)30022-1 30169257

[papt12548-bib-0048] Slater, J. , & Liddiard, K. (2018). Why disability studies scholars must challenge transmisogyny and transphobia. Canadian Journal of Disability Studies, 7(2), 83–93. 10.15353/cjds.v7i2.424

[papt12548-bib-0049] Smith, J. A. , Flowers, P. , & Larkin, M. (2013). Interpretative phenomenological analysis: Theory, method and research. Sage.

[papt12548-bib-0050] Strauss, P. , Cook, A. , Winter, S. , Watson, V. , Wright Toussaint, D. , & Lin, A. (2017). Trans pathways: The mental health experiences and care pathways of trans young people. Summary of results. Telethon Kids Institute. Retrieved from. https://www.telethonkids.org.au/globalassets/media/documents/brain‐‐behaviour/trans‐pathwayreport‐web.pdf

[papt12548-bib-0051] Telfer, M. M. , Tollit, M. A. , Pace, C. C. , & Pang, K. C. (2018). Australian standards of care and treatment guidelines for transgender and gender diverse children and adolescents. Medical Journal of Australia, 209, 132–136. 10.5694/mja17.01044 29902964

[papt12548-bib-0052] Town, R. , Hayes, D. , Fonagy, P. , & Stapley, E. (2022). A qualitative investigation of LGBTQ+ young people's experiences and perceptions of self‐managing their mental health. European Child & Adolescent Psychiatry, 31(9), 1441–1454. 10.1007/s00787-021-01783-w 33903961 PMC8075021

[papt12548-bib-0053] WHO . (2021). Mental health of adolescents. https://www.who.int/news‐room/fact‐sheets/detail/adolescent‐mental‐health

[papt12548-bib-0054] Williams, A. J. , Jones, C. , Arcelus, J. , Townsend, E. , Lazaridou, A. , & Michail, M. (2021). A systematic review and meta‐analysis of victimisation and mental health prevalence among LGBTQ+ young people with experiences of selfharm and suicide. PLoS One, 16(1), e0245268. 10.1371/journal.pone.0245268 33481862 PMC7822285

[papt12548-bib-0055] Williams, D. Y. , Hall, W. J. , Dawes, H. C. , Srivastava, A. , Radtke, S. R. , Ramon, M. , Bouchard, D. , Chen, W. T. , & Goldbach, J. T. (2023). Relationships between internalized stigma and depression and suicide risk among queer youth in the United States: A systematic review and meta‐analysis. Frontiers in Psychiatry, 14, 1205581. 10.3389/fpsyt.2023.1205581 37547195 PMC10399219

[papt12548-bib-0056] Wittgens, C. , Fischer, M. M. , Buspavanich, P. , Theobald, S. , Schweizer, K. , & Trautmann, S. (2022). Mental health in people with minority sexual orientations: A meta‐analysis of population‐based studies. Acta Psychiatrica Scandinavica, 145(4), 357–372. 10.1111/acps.13405 35090051

[papt12548-bib-0057] Zeeman, L. , Aranda, K. , Sherriff, N. , & Cocking, C. (2017). Promoting resilience and emotional well‐being of transgender young people: Research at the intersections of gender and sexuality. Journal of Youth Studies, 20, 382–397. 10.1080/13676261.2016.1232481

